# Supplementary material for: Pyruvate Dehydrogenase Kinase 1 inhibition mediated oxidative phosphorylation enhancement in cartilage promotes osteoarthritis progression
Source: BMC Musculoskelet Disord. 2023 Jul 20;24:597. doi: 10.1186/s12891-023-06585-6 (PMC10357736; doi:10.1186/s12891-023-06585-6)
Supplement: Supplementary file 1 — Additional file 1. [file 12891_2023_6585_MOESM1_ESM.zip › BP.Gsea.1653623667859/GOBP_DEOXYRIBOSE_PHOSPHATE_METABOLIC_PROCESS.html]

Details for gene set GOBP\_DEOXYRIBOSE\_PHOSPHATE\_METABOLIC\_PROCESS[GSEA]

|  || Dataset | 55457\_collapsed\_to\_symbols.cls.cls #OA\_versus\_normal.cls.cls #OA\_versus\_normal\_repos |
| Phenotype | cls.cls#OA\_versus\_normal\_repos |
| Upregulated in class | OA |
| GeneSet | GOBP\_DEOXYRIBOSE\_PHOSPHATE\_METABOLIC\_PROCESS |
| Enrichment Score (ES) | 0.42466712 |
| Normalized Enrichment Score (NES) | 1.5955989 |
| Nominal p-value | 0.017326733 |
| FDR q-value | 0.27393025 |
| FWER p-Value | 1.0 |
Table: GSEA Results Summary

  

Fig 1: Enrichment plot: GOBP\_DEOXYRIBOSE\_PHOSPHATE\_METABOLIC\_PROCESS      
 Profile of the Running ES Score & Positions of GeneSet Members on the Rank Ordered List

  

| SYMBOL | TITLE | RANK IN GENE LIST | RANK METRIC SCORE | RUNNING ES | CORE ENRICHMENT || 1 | DGUOK | deoxyguanosine kinase [Source:HGNC Symbol;Acc:HGNC:2858] | 96 | 0.917 | 0.0695 | Yes |
| 2 | ADK | adenosine kinase [Source:HGNC Symbol;Acc:HGNC:257] | 127 | 0.878 | 0.1408 | Yes |
| 3 | NUDT18 | nudix hydrolase 18 [Source:HGNC Symbol;Acc:HGNC:26194] | 233 | 0.797 | 0.1995 | Yes |
| 4 | OGG1 | 8-oxoguanine DNA glycosylase [Source:HGNC Symbol;Acc:HGNC:8125] | 453 | 0.681 | 0.2396 | Yes |
| 5 | DPYS | dihydropyrimidinase [Source:HGNC Symbol;Acc:HGNC:3013] | 509 | 0.656 | 0.2903 | Yes |
| 6 | DCK | deoxycytidine kinase [Source:HGNC Symbol;Acc:HGNC:2704] | 920 | 0.537 | 0.3034 | Yes |
| 7 | DERA | deoxyribose-phosphate aldolase [Source:HGNC Symbol;Acc:HGNC:24269] | 975 | 0.523 | 0.3431 | Yes |
| 8 | SMUG1 | single-strand-selective monofunctional uracil-DNA glycosylase 1 [Source:HGNC Symbol;Acc:HGNC:17148] | 1068 | 0.501 | 0.3780 | Yes |
| 9 | DCTD | dCMP deaminase [Source:HGNC Symbol;Acc:HGNC:2710] | 1107 | 0.494 | 0.4165 | Yes |
| 10 | GUK1 | guanylate kinase 1 [Source:HGNC Symbol;Acc:HGNC:4693] | 1640 | 0.407 | 0.4093 | Yes |
| 11 | NT5C | "5', 3'-nucleotidase, cytosolic [Source:HGNC Symbol;Acc:HGNC:17144]" | 1852 | 0.379 | 0.4247 | Yes |
| 12 | NTHL1 | nth like DNA glycosylase 1 [Source:HGNC Symbol;Acc:HGNC:8028] | 3219 | 0.228 | 0.3377 | No |
| 13 | RRM2 | ribonucleotide reductase regulatory subunit M2 [Source:HGNC Symbol;Acc:HGNC:10452] | 3505 | 0.202 | 0.3324 | No |
| 14 | DTYMK | deoxythymidylate kinase [Source:HGNC Symbol;Acc:HGNC:3061] | 3538 | 0.199 | 0.3466 | No |
| 15 | TYMP | thymidine phosphorylase [Source:HGNC Symbol;Acc:HGNC:3148] | 3588 | 0.195 | 0.3592 | No |
| 16 | TBPL1 | TATA-box binding protein like 1 [Source:HGNC Symbol;Acc:HGNC:11589] | 3794 | 0.178 | 0.3581 | No |
| 17 | DPYD | dihydropyrimidine dehydrogenase [Source:HGNC Symbol;Acc:HGNC:3012] | 3830 | 0.173 | 0.3699 | No |
| 18 | AK5 | adenylate kinase 5 [Source:HGNC Symbol;Acc:HGNC:365] | 3841 | 0.172 | 0.3836 | No |
| 19 | DCTPP1 | dCTP pyrophosphatase 1 [Source:HGNC Symbol;Acc:HGNC:28777] | 4044 | 0.152 | 0.3806 | No |
| 20 | TYMS | thymidylate synthetase [Source:HGNC Symbol;Acc:HGNC:12441] | 4328 | 0.126 | 0.3692 | No |
| 21 | RRM1 | ribonucleotide reductase catalytic subunit M1 [Source:HGNC Symbol;Acc:HGNC:10451] | 4501 | 0.113 | 0.3653 | No |
| 22 | SAMHD1 | SAM and HD domain containing deoxynucleoside triphosphate triphosphohydrolase 1 [Source:HGNC Symbol;Acc:HGNC:15925] | 5790 | 0.009 | 0.2659 | No |
| 23 | DUT | deoxyuridine triphosphatase [Source:HGNC Symbol;Acc:HGNC:3078] | 5981 | -0.003 | 0.2514 | No |
| 24 | UNG | uracil DNA glycosylase [Source:HGNC Symbol;Acc:HGNC:12572] | 6078 | -0.011 | 0.2448 | No |
| 25 | NT5M | "5',3'-nucleotidase, mitochondrial [Source:HGNC Symbol;Acc:HGNC:15769]" | 7781 | -0.140 | 0.1243 | No |
| 26 | ADA | adenosine deaminase [Source:HGNC Symbol;Acc:HGNC:186] | 8105 | -0.163 | 0.1129 | No |
| 27 | NUDT15 | nudix hydrolase 15 [Source:HGNC Symbol;Acc:HGNC:23063] | 8305 | -0.178 | 0.1124 | No |
| 28 | XDH | xanthine dehydrogenase [Source:HGNC Symbol;Acc:HGNC:12805] | 8366 | -0.183 | 0.1231 | No |
| 29 | UPB1 | beta-ureidopropionase 1 [Source:HGNC Symbol;Acc:HGNC:16297] | 9609 | -0.284 | 0.0503 | No |
| 30 | PNP | purine nucleoside phosphorylase [Source:HGNC Symbol;Acc:HGNC:7892] | 9657 | -0.288 | 0.0708 | No |
| 31 | SHMT1 | serine hydroxymethyltransferase 1 [Source:HGNC Symbol;Acc:HGNC:10850] | 9727 | -0.294 | 0.0901 | No |
| 32 | TDG | thymine DNA glycosylase [Source:HGNC Symbol;Acc:HGNC:11700] | 10728 | -0.396 | 0.0456 | No |
| 33 | NEIL1 | nei like DNA glycosylase 1 [Source:HGNC Symbol;Acc:HGNC:18448] | 10963 | -0.425 | 0.0631 | No |
| 34 | MBD4 | "methyl-CpG binding domain 4, DNA glycosylase [Source:HGNC Symbol;Acc:HGNC:6919]" | 11114 | -0.445 | 0.0888 | No |
| 35 | UPP1 | uridine phosphorylase 1 [Source:HGNC Symbol;Acc:HGNC:12576] | 12013 | -0.595 | 0.0689 | No |
Table: GSEA details [plain text format]

  

Fig 2: GOBP\_DEOXYRIBOSE\_PHOSPHATE\_METABOLIC\_PROCESS      
 Blue-Pink O' Gram in the Space of the Analyzed GeneSet

  

Fig 3: GOBP\_DEOXYRIBOSE\_PHOSPHATE\_METABOLIC\_PROCESS: Random ES distribution      
 Gene set null distribution of ES for **GOBP\_DEOXYRIBOSE\_PHOSPHATE\_METABOLIC\_PROCESS**
